# Nf2/Merlin: a coordinator of receptor signalling and intercellular contact

**DOI:** 10.1038/sj.bjc.6604002

**Published:** 2007-10-30

**Authors:** M Curto, A I McClatchey

**Affiliations:** 1 Massachusetts General Hospital Center for Cancer Research and Department of Pathology, Harvard Medical School, 149 13th Street, Charlestown, MA 02129, USA

**Keywords:** Merlin, Ezrin, EGFR, contact-inhibition, adherens junctions, cytoskeleton

## Abstract

This review explores possible mechanisms by which the neurofibromatosis type-2 tumour suppressor Merlin regulates contact-dependent inhibition of proliferation. Starting from an evolutionary perspective, the concurrent emergence of intercellular contacts and proliferation control in multicellular organisms is first considered. Following a brief survey of the molecular and subcellular milieus in which merlin performs its function, the importance of different cellular and biological contexts in defining the function of merlin is discussed. Finally, an integrated model for merlin and the Ezrin, Radixin, and Moesin (ERM) proteins functioning in the regulation of cellular interfaces is proposed.

All cancers result from the cumulative acquisition of molecular defects within a basic core of cellular functions that control proliferation, apoptosis, and invasiveness (Hanahan and Weinberg, 2000). Regardless of the specific alterations, their combined effects must also disable the mechanism of contact-dependent inhibition of proliferation, namely the cell's ability to prevent activation of the proliferation machinery in response to contact with neighbouring cells. Indeed, loss of contact-dependent inhibition of proliferation is an absolute *conditio sine qua non* for the development and progression of any solid tumour. This phenomenon was first described in the early 1960s with the observation that normal cultured diploid cells can reach a maximum saturation density beyond which cell number and DNA synthesis are unaffected by media replenishment (Levine *et al*, 1965). This finding also introduced the idea that upon adhering to each other, cells can enter and maintain a quiescent state despite the unlimited availability of nutrients and growth-promoting factors – a phenomenon that occurs *in vivo* in all adult solid tissues. Conversely, the uncoupling of mitogenic signals from the blockage imposed by cell:cell adhesion is a physiological event during embryogenesis and tissue homoeostasis and regeneration. Therefore, highly regulated molecular machinery must exist to conditionally establish and resolve the link between intercellular contacts and mitogenic signalling. Although still poorly understood, the molecular mechanisms that coordinate intercellular adhesion and cell proliferation are beginning to emerge (Brunton *et al*, 2004). A detailed understanding of the molecular components and events that govern this phenomenon will be essential to understanding and intercepting the process of tumourigenesis.

Mounting evidence indicates that the neurofibromatosis type-2 (NF2) tumour suppressor, Merlin (also known as schwannomin) is a critical regulator of contact-dependent inhibition of proliferation. Mutations in the *NF2* tumour suppressor gene underlie the familial cancer syndrome NF2, which is characterised by the development of tumours of the nervous system such as bilateral vestibular schwannomas and meningiomas (Baser *et al*, 2002). Biallelic *NF2* inactivation also underlies the development of many sporadic schwannomas and meningiomas, as well as malignant mesothelioma, an essentially untreatable cancer. Despite the restricted spectrum of tumours associated with loss of NF2, Merlin is a widely expressed protein and studies from several model organisms have shown that Merlin plays an important role in the function of many different cell types (McClatchey and Giovannini, 2005). Although its molecular function has been elusive, recent studies suggest that Merlin can coordinate the establishment of intercellular contacts with the concomitant suppression of mitogenic signals at the membrane.

## EVOLUTION

The transition from prokaryotes to early unicellular eukaryotes was accompanied by the generation of a variety of membrane subdomains including cortical membrane patches, protrusions and ‘landmarks’ utilised during locomotion, budding, or conjugation. This increased complexity in cellular polarisation was likely facilitated by the acquisition of flexible membranes and a primordial cytoskeleton (endoskeleton), together with the emergence of small GTPases that enabled directional (exo- and endocytic) membrane flow, and localised cytoskeleton assembly (Jekely, 2003; Nelson, 2003). On the eve of multicellular life, unicellular polarity had evolved a sophisticated membrane complexity with specialised subdomains of disparate forms and functions such as cilia, flagella, cytostomata, and pseudopodia. Unicellular choanoflagellates, the most likely cenancestor of metazoa, already utilised adhesion machineries for food capture or colony formation, and transmembrane receptors that can respond to environmental cues. In fact, it has been recently discovered that proteins (spectrin, cadherins, G protein-coupled receptors) and interaction motifs (SH2, EGF) thought to be unique to metazoa were already in place and cell proliferation was already under the control of receptor and non-receptor tyrosine kinases (King *et al*, 2003; King, 2004).

Unicellular organisms were capable of organizing their membranes and pacing proliferation in response to extracellular signals, but the transition to multicellularity necessitated the structural and functional coordination of these membrane subdomains at the interface between cells. This might have occurred via the molecular rewiring of existing adhesive and transmembrane signalling apparata utilising intracellular proteins/modules capable of orchestrating multiple interactions at the plasma membrane. Such a function could have been carried out by scaffolding proteins containing, for example, PDZ and LIM domains but these were already found in prokaryotes and likely regulated polarity (Te Velthuis *et al*, 2007). The further increase in membrane complexity inherent in the transition to multicellularity was accompanied by the emergence of new protein domains, several including the four-point-one, ezrin, radixin, moesin (FERM) domain. Indeed, 14 orthologous groups of FERM domain-containing proteins can be identified in worms, flies, and mammals suggesting that a dramatic expansion of this superfamily occurred in the earliest metazoa (Bretscher *et al*, 2002). The trilobed FERM domain is generally thought to mediate membrane association and can be tethered to a number of different functional domains. In a subset of proteins, including Merlin and the ERM proteins, the FERM domain is appended to modules that link it to the cytoskeleton, and poise these proteins to coordinate multiple signalling events at the membrane with specific architectural features of the cell. Thus, the FERM domains appear to have evolved within an already polarised cellular context to further organise membrane domains in response to the requirements imposed by multicellularity.

Nutrient availability is the major regulator of proliferation in unicellular organisms; under conditions of unlimited availability proliferation appears to be the default. The complex morphogenetic processes and compartmentalised tissue functions that evolved in metazoa necessitated the ability to inhibit cell proliferation, effectively disengaging the proliferative machinery from that which senses and responds to surrounding conditions (Edgar, 2006). Among FERM domain-containing proteins, the function of the tumour suppressor Merlin is most clearly linked to the inhibition of cell proliferation. In fact, accumulating evidence suggests that Merlin physically and functionally links cell:cell communication with negative regulation of proliferation, a conclusion drawn from molecular, cellular, and biological studies (reviewed in McClatchey and Giovannini, 2005).

## MOLECULAR-CELLULAR CONTEXT

Studies of the closely related ERM proteins have made important contributions to our understanding of the molecular function of merlin. The term ‘membrane:cytoskeleton linker’ used to describe both Merlin and the ERM proteins highlights the bipartite subcellular context in which they operate. A standard view of ERM function depicts cycling between ‘active’ and ‘inactive’ states and considers ‘activity’ to be the tethering of certain membrane proteins to the cytoskeleton (Bretscher *et al*, 2002). Although studies of Merlin yield many parallels, it is increasingly apparent that more sophisticated models of Merlin (and ERM) function must be considered. More refined models would consider the possibility that Merlin can exist in more than two states and can bring together and regulate multiple partners. Indeed, an increasing number of proteins have been reported to physically interact with Merlin and an increasing number of functional activities have been ascribed to Merlin (McClatchey and Giovannini, 2005). Collectively, these studies suggest a physical and functional role for Merlin in regulating cell:cell adhesion, transmembrane signalling receptors, Rho-GTPase signalling, and in modulating actin cytoskeleton dynamics. Could Merlin do all this?

### The membrane

Most studies conclude that membrane association is necessary for the growth-suppressing function of Merlin. Mutant versions of Merlin that cannot localise to the membrane cannot inhibit cell proliferation. In addition, Merlin can associate with several membrane proteins, including both adhesion receptors and membrane receptors that regulate cell proliferation and differentiation.

In normal cells, Merlin is recruited to cadherin-containing complexes at nascent cell:cell contacts and is regulated by cell:cell adhesion. Conversely, primary *Nf2*^−/−^ cells of several types do not form stable adherens junctions (AJs) (Lallemand *et al*, 2003). Merlin has also been reported to associate with other types of adhesion receptors, including the cell:extracellular matrix receptor CD44 and *β*1-integrin, perhaps reflecting alternative ways for merlin to coordinate the receipt of information from the extracellular environment with the proliferative machinery (Morrison *et al*, 2001; Fernandez-Valle *et al*, 2002). Indeed, in some cells the association of Merlin with CD44 has been shown to be required for contact-dependent inhibition of proliferation (Morrison *et al*, 2001).

**Figure 1:**
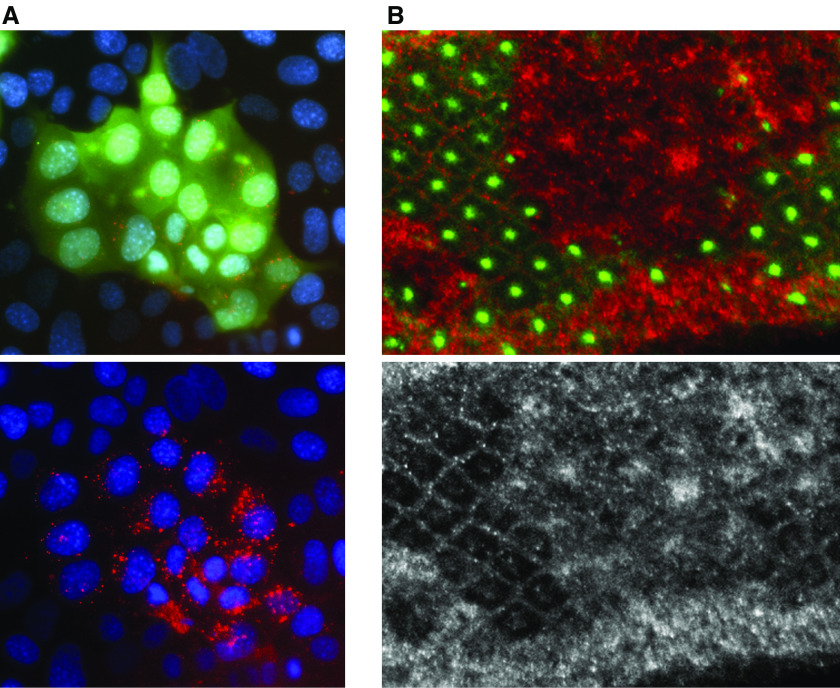
Merlin differentially controls the membrane distribution of EGFR in mammalian *vs* insect cells. (**A**) Mosaic culture of wild-type mouse epithelial cells surrounding a clone of *Nf2*-deficient cells marked with a green fluorescent protein-expressing adenovirus (green). Upon stimulation (30 min), fluorescent EGF (Texas Red conjugated) is internalised and concentrated within intracellular vesicles in cells that do not express Merlin, but not in wild-type cells (Curto *et al*, 2007). Bottom panel, Texas Red EGF. (**B**) *Drosophila* eye imaginal disc. Mutant clones containing mutations in both merlin and Expanded are identified by the loss of Merlin staining (green). In these cells, the steady-state levels of EGFR are increased (Maitra *et al*, 2006) (figure kindly provided by Sushmita Maitra and Richard Fehon, University of Chicago). Bottom panel, EGFR.

Recent studies suggest that Merlin directly controls the surface availability and function of membrane receptors that regulate proliferation and differentiation in both *Drosophila* and mammalian cells (Figure 1). In the fly, loss of both Merlin and the related tumour suppressor, Expanded, leads to increased surface levels and altered distribution of certain membrane receptors including epidermal growth factor receptor (EGFR), Notch, Patched, and Fat, suggesting that Merlin normally promotes the clearance of receptors from the plasma membrane. This was shown to be accompanied by increased signalling output for some receptors (Maitra *et al*, 2006). In cultured mammalian cells, Merlin inhibits the internalisation, effector complexing, and downstream signalling of activated EGFR upon cell:cell contact, consistent with the idea that Merlin normally sequesters EGFR into a non-signalling plasma membrane compartment; as follows, proliferation and EGFR activation are inhibited at high cell density in wild-type but not *Nf2*^−/−^ cells (Curto *et al*, 2007). Although these two studies seem to reach differing views of how Merlin regulates membrane receptor surface availability/function – in one case promoting turnover and the other stabilising surface receptors – it is possible that the primary function of Merlin in both cases is to retain receptors in a certain membrane compartment and that the consequence of that is dependent upon species, cell context, cell type or even the identity of the receptor itself. Alternatively, these differences could reflect the concomitant loss of Expanded in the fly studies (Maitra *et al*, 2006).

**Figure 2:**
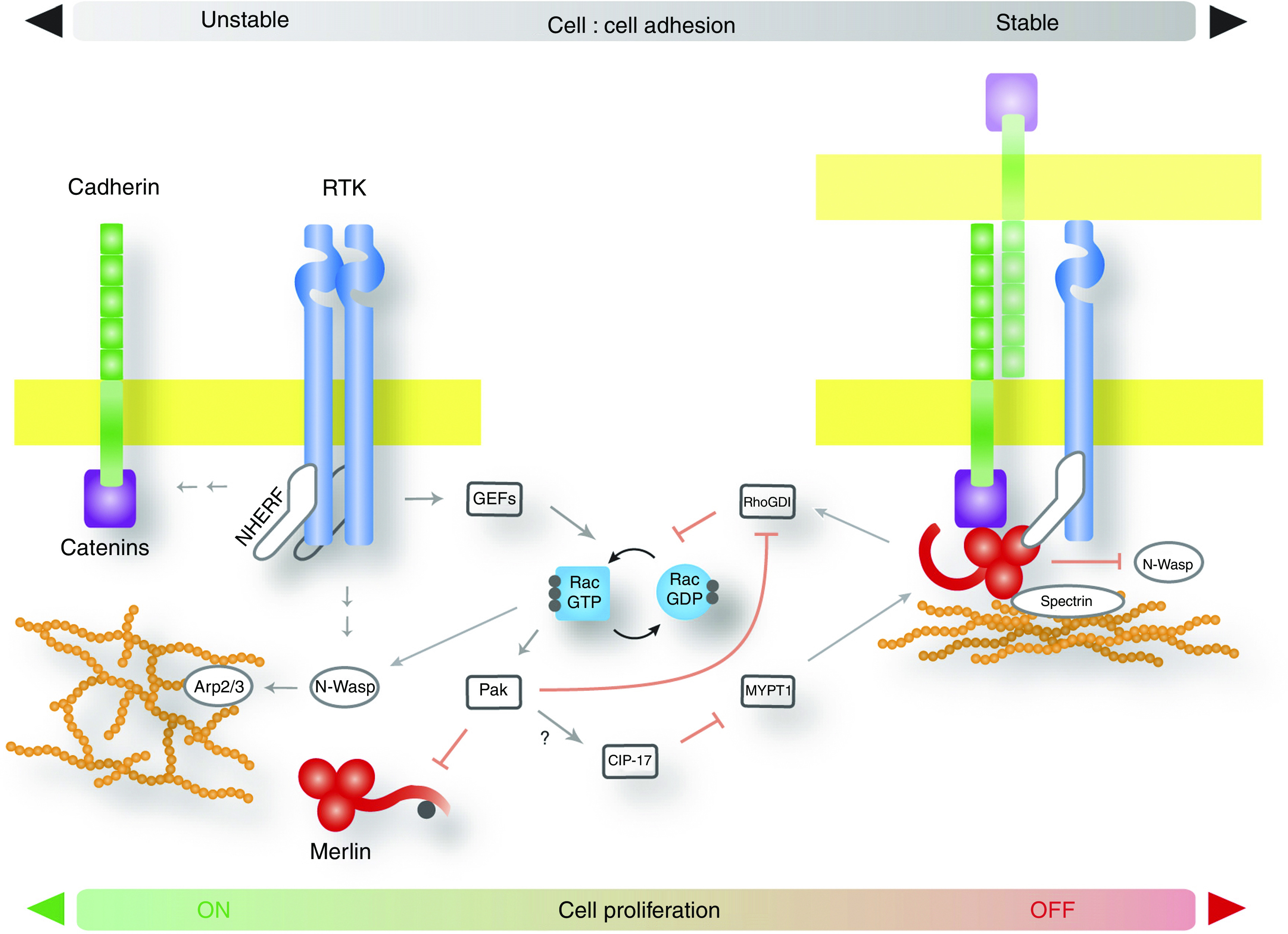
Merlin coordinates membrane receptor signalling and cell:cell contact. Schematic depiction of the basic molecular circuitry utilised by Merlin to link intercellular adhesion with the regulation of membrane receptor signalling. As an example, the relationships among Merlin, Cadherin and the mitogenic receptor tyrosine kinase (RTK) EGFR, are shown here. (Left) In conditions where cell:cell contacts are minimal or unstable, such as remodelling tissues or sparse cultured cells, signals from activated RTKs lead to instability of cadherin-catenin-containing complexes, highly dynamic actin cytoskeleton, and increased activation of Rac which, in turn, keeps Merlin inactive. Proliferative signals are transduced unopposed. (Right) The progressive recruitment of Merlin to newly formed cell:cell contacts is accompanied by a decline in Rac activity, decreased turnover of the cortical actin cytoskeleton, and AJ stabilisation. At the same time, complexing of Merlin to EGFR:E-cadherin could concomitantly tether it to the more stable actin network and restrict EGFR to an insoluble adhesive compartment from which it can neither signal nor internalise (see also Figure 1). At late confluence, EGFR signalling induced by serum or exogenous EGF is disabled and proliferative signals are silenced. These events do not occur in the absence of Merlin.

Increasing evidence suggests that Merlin mediates contact-dependent inhibition of proliferation by both sensing cell:cell contact and intercepting mitogenic signalling initiated at the plasma membrane. A simultaneous, contact-dependent complexing of Merlin to both signalling and adhesion receptors would be a simple strategy for accomplishing this (Figure 2). In mammalian cells Merlin associates with EGFR in a contact-dependent manner; this association requires the tandem PDZ domain-containing adaptor Na^+^–H^+^ exchanger regulatory factor 1; ERM-binding protein of 50 kDa (NHE-RF1/EBP50; hereafter referred to as NHE-RF1), which can associate with both Merlin and EGFR (Lazar *et al*, 2004; Curto *et al*, 2007). In fact, NHE-RF1 can also associate with the ERM proteins and, either directly or indirectly with a variety of receptors, including ErbB2 and the platelet-derived growth factor receptor (PDGFR) (James *et al*, 2004; Rangwala *et al*, 2005). The latter is particularly notable given the recent report that Merlin can also negatively regulate signalling downstream of PDGFR (Morrison *et al*, 2007); this study, which did not examine NHE-RF1, concluded that Merlin interfered with PDGFR signalling downstream of its immediate effectors, in contrast to the aforementioned studies of Merlin:EGFR. Notably, however, NHE-RF1 has been reported to associate with internal PDZ binding sites in the EGFR cytoplasmic domain that are in close proximity to the major effector binding sites (Lazar *et al*, 2004); in contrast, NHE-RF1 associates with the extreme C-terminus of PDGFR and therefore may not alter effector association. Interestingly, a role for the closely related NHE-RF2 adaptor in facilitating an association between N-cadherin and PDGFR has recently been reported (Theisen *et al*, 2007). Perhaps the nature of the NHE-RF:receptor association, along with the spatiotemporal specificity achieved by associating with Merlin and/or the ERM proteins, can impose differential physical and signalling regulation upon different membrane receptors. It will be interesting to see whether the association of Merlin with analogous PDZ adaptors is important for receptor surface abundance in *Drosophila*. The output of a variety of receptors could be regulated by Merlin using this general mechanism, potentially explaining the many intracellular pathways that have been reported to be affected by Merlin.

### The actin cytoskeleton

Many studies indicate a functional association between Merlin and the actin cytoskeleton. Biochemically, Merlin can clearly associate with actin *in vitro*. Merlin does not localise to stress fibres and instead, like the ERM proteins, appears to decorate the cortical actin network (James *et al*, 2001). Although lacking the *bona fide* carboxyl-C-terminal actin-binding site found in the ERM proteins, Merlin may instead directly bind F-actin via its N-terminal domain (Brault *et al*, 2001; James *et al*, 2001). Alternatively, indirect tethering of Merlin to the cytoskeleton may occur via actin-binding interactors such as βII-spectrin/fodrin, or heterodimerisation with other ERMs (reviewed in McClatchey and Giovannini, 2005). The importance of actin cytoskeleton association is underscored by the fact that association of Merlin with the cortical actin network is required for growth suppression and inhibition of EGFR signalling (B Cole, MC, and AIM, unpublished data).

A functional association between Merlin and the cortical actin cytoskeleton is also suggested by the fact that loss of merlin is accompanied by disruption of cortical actin structures in some cell types (Bashour *et al*, 2002; Lallemand *et al*, 2003). The disorganised actin cytoskeleton observed in some *Nf2*^−/−^ cells could reflect the ability of Merlin to anchor actin to the membrane, to inhibit signals that maintain a highly dynamic actin cytoskeleton, or both. For example, Merlin can inhibit both Arp2/3- (Manchanda *et al*, 2005) and Rac-induced actin assembly (Pelton *et al*, 1998).

### Regulation

Beyond its physical identity, the membrane:cytoskeleton interface is a dynamic compartment in which Merlin, the ERM proteins, and the molecular circuitry that controls their localisation and function operate. A central node of this circuitry is occupied by the Rho family of small GTPases, well-established regulators of actin cytoskeleton remodelling and membrane trafficking. In contrast to the Rho-dependent activation of the ERM proteins, Rac-dependent phosphorylation of a particular C-terminal serine residue (S518) leads to Merlin inactivation (Shaw *et al*, 2001). This is generally thought to involve the phosphorylation-dependent transition from a ‘closed’ to an ‘open’ conformation; however, while the lack of S518 phosphorylation correlates well with the growth-suppressing function of Merlin, it has not been definitively shown that active Merlin is necessarily ‘closed’. Moreover, Merlin can be phosphorylated at residues other than S518 but the functional consequences of such regulation are not yet known (McClatchey and Giovannini, 2005). Notably, S518 phosphorylation can be reversed by the myosin phosphatase MYPT-1-PP1δ (Jin *et al*, 2006, and references therein). This provides a potentially interesting point of regulatory convergence between Merlin and the ERM proteins given that myosin phosphatase has also been shown to associate with and induce dephosphorylation of the ERM proteins. Another point is provided by recent studies indicating that the Ste-20 kinase Slik can induce phosphorylation of both Merlin and the ERM proteins in the fly (Hughes and Fehon, 2006). Importantly, by analogy to the ability of the ERM proteins to negatively regulate Rho signalling, Merlin can negatively regulate Rac signalling (Shaw *et al*, 2001), perhaps through an association with the Rac regulator Rho-GDI or the Rac effector Pak or by directly controlling membrane association of Rac in some cells (Kissil *et al*, 2002; Okada *et al*, 2005). These core regulatory loops underlie the complex nature of Merlin/ERM-regulated signalling at the membrane:cytoskeleton interface.

## BIOLOGICAL CONTEXTS

The ability of Merlin to physically coordinate multiple signalling entities as described above only hints at the combinatorial level of complexity that is possible, given that Merlin can sense different types of cell contact and regulate multiple membrane receptors. The outcome of this coordinated regulation is likely to be context dependent *in vivo*. The exquisite requirement for regulating cell:cell contact and proliferation during tissue morphogenesis in both developmental and regenerative processes is likely to make these contexts critically dependent upon Merlin function.

For example, *Nf2* is an essential gene in *Drosophila* since null mutations result in embryonic lethality. Hypomorphic mutants display tissue overgrowth consistent with its anti-proliferative function. Recent studies have used clonal deletion/somatic recombination in developing imaginal discs to generate cells lacking both Merlin and the related tumour suppressor Expanded (*Mer/Ex*). The hyperplastic outgrowth of the mutant clones in several adult structures (eye, wing, leg) appears to result from the combined effect of increased proliferation and reduced apoptosis during development of these structures and is associated with increased surface abundance of several signalling and adhesion receptors (Edgar, 2006; Maitra *et al*, 2006). In this regard, persistent EGFR signalling in *Mer/Ex* mutants is likely to antagonise normal apoptosis-inducing signals within the developing imaginal discs and results in increased cell survival (Yang and Baker, 2003).

Signalling via the recently characterised Salvador–Warts–Hippo (SWH) pathway is also elevated in *Mer/Ex* double mutant cells (Edgar, 2006; Harvey and Tapon, 2007). It has been proposed that Merlin and Expanded are upstream regulators of the SWH pathway, which suppresses Yki-dependent transcription of CycE, DIAP, and *Mer/Ex* themselves. Direct molecular links between Merlin and components of this pathway have not yet been found and the identity of the upstream signal(s) that are regulated by *Mer/Ex* is still unknown. The extent of these phenotypes might also reflect a synergistic effect of the combined *Mer/Ex* mutation. However, consistent with the theme derived from studies of cultured mammalian cells, the adhesion molecule Fat appears to confer contact-dependent regulation of the pathway (Harvey and Tapon, 2007).

Merlin is also required for embryonic development in zebrafish, as embryos fail to develop after anti-sense-mediated inactivation of the *nf2* gene. Loss-of-function *nf2* mutants display biliary hyperplasia and formation of choledochal cysts indicating that Merlin is an important regulator of biliary tract development in zebrafish (Sadler *et al*, 2005). These findings complement the previous observations that both hepatocellular and cholangiocellular neoplasia develop in the liver of *Nf2* heterozygous mutant mice (McClatchey *et al*, 1998).

The generation of *Nf2*-mutant mouse models has contributed important information regarding Merlin function in development and tumourigenesis. Merlin is required for the normal development of several tissues; *Nf2*-null embryos fail to gastrulate due to extra-embryonic defects (McClatchey and Giovannini, 2005). This defect is rescued in mosaic *Nf2*^*+/*^↔*Nf2*^*−/−*^ embryos which go on to develop a number of additional abnormalities, including in heart and skeletal development and in neural tube closure (AIM unpublished data). In fact, recent studies have shown that targeted deletion of *Nf2* in the developing nervous system causes neural tube defects from impaired tissue fusion (McLaughlin *et al*, 2007). Abnormal cell detachment and apoptosis are found at the leading front of unfused folds and the apical side of ventricular neuroepithelia. These phenotypes appear to derive from the inability to form apico-junctional complexes (McLaughlin *et al*, 2007), consistent with the role of Merlin in controlling formation and stabilisation of AJs (Lallemand *et al*, 2003).

Heterozygous *Nf2*^*+/−*^-mutant mice develop a spectrum of unusually highly metastatic tumours, mainly osteosarcomas, fibrosarcomas, and liver carcinomas, that exhibit loss of the wild-type allele. Although these mice do not spontaneously develop schwannomas, meningiomas, or mesotheliomas, mouse models for each have been developed through the use of conditional mutant *Nf2* alleles (Giovannini *et al*, 2000). Altogether, these studies from different organisms and biological settings reveal that perturbing Merlin function yields dramatic consequences over a biological continuum from development to cancer.

## PERSPECTIVES

In simple two-dimensional cell culture models the establishment of cell:cell contact is progressive. Despite a similar morphological appearance, the cellular physiologies of early *vs* late confluent cultures are remarkably different and signalling that occurs in non-contacting cells can still operate in early but not late stages of confluence (Rothen-Rutishauser *et al*, 1998). Silencing of proliferative signals from the time of initial closure of a monolayer requires a few days to complete (Curto *et al*, 2007). Indeed, our understanding of membrane receptor signalling in cultured cells may be frequently confounded by inadequately defined cell densities. An example of how Merlin could mediate the progressive coordination of cadherin-based contact with the inhibition of EGFR signalling is presented in the model in Figure 2. By operating in a reverse fashion, this molecular circuitry could also control the disassembly of cell:cell contacts in response to morphogenetic or regenerative stimuli. For example, receptor and/or non-receptor tyrosine kinase activity can destabilise cell adhesions directly by phosphorylating cadherin-catenin complexes (Brunton *et al*, 2004), or indirectly by promoting Rac/Pak-mediated inactivation of Merlin (Kissil *et al*, 2002; Shaw *et al*, 2001). In this scenario, Merlin could serve as a three-channel rheostat linking, (1) the metering of lateral surface area engaged in intercellular contacts, to (2) the localised modulation of specific signalling receptors at the interfacing membranes, to (3) the tensegrity of the cortical actin cytoskeleton. Thresholds of receptor activity could be affected by normal or pathological changes in the stoichiometry of these interactions. Through association with the cortical cytoskeleton and a reciprocal functional relationships with Rho-GTPases that regulate actomyosin contractility, Merlin and the ERM proteins seem poised to play an important role in linking changes in cellular tensegrity to mitogenic signalling output.

Increasing evidence suggest that the function of Merlin in stabilising AJs could be opposed by that of the ERM proteins. Despite their similarity, the ERM proteins cannot compensate for Merlin loss and *vice versa*; moreover, Merlin and the ERM proteins can form heterodimers and exhibit overlapping but distinct membrane localisations. In mature epithelia, the ERM proteins are largely restricted to the apical domain where they are required for proper localisation and regulation of apical surface proteins, including NHE-RF1 itself (reviewed in Fievet *et al*, 2007). The coexistence of contacting (junctional) and non-contacting (apical) surfaces within the same epithelial cell necessitates mechanisms for forming, maintaining, and reversing these compartments. In this context, Merlin and the ERM proteins could play opposing functions to partition junctional and apical membrane domains in mature epithelia.

**Figure 3:**
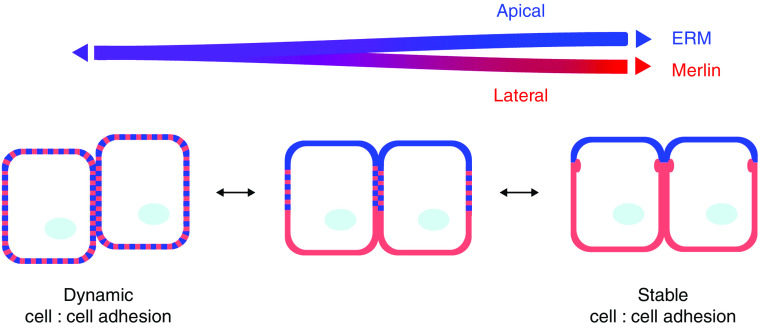
Merlin and the Ezrin, Radixin, and Moesin (ERM) proteins as regulators of cellular interfaces. In multicellular organisms, a regulated instability of specific intercellular contacts is required during morphogenesis and tissue regeneration. While normally localised in the apical domain of stable epithelia (right), the ERM protein Ezrin can also be found within dynamic cell:cell contacts (left), where Merlin also localises. As contacts stabilise to form mature epithelia, this temporary intermixing of Merlin and Ezrin at membrane surfaces is resolved upon their partitioning into junctional and apical domains, respectively (right). Given the prominent role of the ERM proteins in maintaining organised apical surfaces (ie plasma membrane domains devoid of intercellular contacts), and that of Merlin in stabilising cell:cell contacts, localised intermixing could counteract the function of each, promoting a dynamic state of intercellular adhesion. Ezrin, blue; Merlin, red.

Interestingly, a series of *in vitro* studies also support a role for active Ezrin in destabilising cell contacts (Pujuguet *et al*, 2003; Elliott *et al*, 2004, 2005). Loss-of-function studies in *Caenorhabditis elegans*, *Drosophila*, and mouse reveal defects specifically in morphogenetic processes involving epithelial remodelling and dynamic instability of intercellular contacts (Fievet *et al*, 2007). These studies suggest that the ERM proteins are necessary for proper AJ remodelling during epithelial morphogenesis. Notably, in some developing epithelia, including the mouse blastomere, intestinal epithelium and epidermis, Ezrin is transiently and markedly concentrated along cell:cell boundaries where Merlin normally localises (Dard *et al*, 2004; Saotome *et al*, 2004; A Gladden and AIM, unpublished data). An intriguing possibility is that the coexistence of Merlin and Ezrin within the same membrane domain could maintain a dynamic state of the cell:cell interface (Figure 3). This could be particularly important in proliferating epithelial tissues where morphogenetic processes involving the establishment or remodelling of cell contacts are coordinated with proliferative and differentiation signals. The progressive partitioning of these proteins into junctional and apical domains, respectively, would then yield a mature stable epithelium. Given the prominent role of ERM proteins in maintaining organised apical surfaces (ie plasma membrane domains devoid of intercellular contacts), and that of Merlin in stabilising cell:cell contacts, localised intermixing could counteract the function of each, promoting a dynamic state of intercellular adhesion. A compelling parallel is provided by recent studies linking Ezrin over-expression to tumour metastasis – a fundamental property of which is junctional instability (Khanna *et al*, 2004; Yu *et al*, 2004; Elliott *et al*, 2005).

While more detailed investigations are necessary to verify such a model, an essential function of Merlin and the ERM proteins may be to work in concert to physically and functionally regulate the cellular interfaces of metazoa. This possibility has also a fascinating, yet unsettling, implication: that cancers may arise from deregulating the very same mechanisms upon which multicellular life is built.
